# Ethanol Microsensors with a Readout Circuit Manufactured Using the CMOS-MEMS Technique

**DOI:** 10.3390/s150101623

**Published:** 2015-01-14

**Authors:** Ming-Zhi Yang, Ching-Liang Dai

**Affiliations:** Department of Mechanical Engineering, National Chung Hsing University, Taichung 402, Taiwan; E-Mail: d099061005@mail.nchu.edu.tw

**Keywords:** ethanol sensor, tin dioxide film, heater, CMOS-MEMS

## Abstract

The design and fabrication of an ethanol microsensor integrated with a readout circuit on-a-chip using the complementary metal oxide semiconductor (CMOS)-microelectro-mechanical system (MEMS) technique are investigated. The ethanol sensor is made up of a heater, a sensitive film and interdigitated electrodes. The sensitive film is tin dioxide that is prepared by the sol-gel method. The heater is located under the interdigitated electrodes, and the sensitive film is coated on the interdigitated electrodes. The sensitive film needs a working temperature of 220 °C. The heater is employed to provide the working temperature of sensitive film. The sensor generates a change in capacitance when the sensitive film senses ethanol gas. A readout circuit is used to convert the capacitance variation of the sensor into the output frequency. Experiments show that the sensitivity of the ethanol sensor is 0.9 MHz/ppm.

## 1. Introduction

Ethanol sensors can be applied in environmental and industrial monitoring. Humans can inhale over 1000 ppm ethanol vapor that can cause headaches, nausea, balance disorders, dizziness and confusion [1]. Thereby, ethanol sensors play an important role in avoiding the possible damages caused by ethanol. Traditional sensors have the disadvantages of large volume and high cost. On the contrary, microsensors have the benefits of high performance, small volume, easy mass-production and low cost [2,3]. Various traditional sensors have been miniaturized as microsensors using MEMS technology [4–6]. Wan *et al.* [7] used MEMS technology to fabricate an ethanol microsensor on a silicon-based membrane. The sensitive film of the ethanol sensor was ZnO nanowire, and the material needed a working temperature of 300 °C to sense ethanol gas. To provide the working temperature, a micro heater was designed under the sensitive film. A temperature sensor was designed to monitor the heater temperature. With the same sensitive material, Nguyen *et al.* [8] developed a gas microsensor on the silicon dioxide membrane using MEMS technology. To enhance the sensitivity of the sensor, the Au islands as catalyst material were combined with the sensitive film. The response of the gas sensor increased to 125% at 250 ppm ethanol. The working temperature of the film was 450 °C, so the power consumption of the sensor was as high as 4 W. The power consumption of the gas sensor depended on the working temperature of the sensitive film. Pandya *et al.* [9] proposed an ethanol microsensor with a low working temperature sensitive film. The sensitive material of ZnO nanostructure was prepared, and its working temperature was 100 °C, which reduced the power consumption. The sensitive material of the ethanol sensors [7–9] were ZnO. The sensitivity of gas sensor depends on its sensitive material. Therefore, in this work we prepare tin dioxide nanostructure as the sensitive ethanol material to enhance the sensitivity of ethanol sensor. In addition, these sensors [7–9] were not integrated with circuitry on-a-chip. The integrated sensors with circuits have the advantages of low interference, high performance and low packaging cost. In this study, we develop an integrated ethanol microsensor with a readout circuit on-a-chip.

The use of the commercial CMOS process to develop MEMS devices is called the CMOS-MEMS technique [10–12]. This technique has been employed to fabricate various microsensors and microactuators [13–15]. Microsensors manufactured by this technique can integrate with readout circuits on-a-chip [16–18]. Liao *et al.* [19] proposed an ethanol microsensor with an inverting amplifier circuit fabricated using the commercial 0.18 μm CMOS process. The sensitive material of the ethanol sensor was zinc oxide, and the sensor was a resistive type. The inverting amplifier circuit converted the resistance of the sensor into the output voltage. The experiments showed that the smallest sensing concentration of the sensor was about 50 ppm ethanol. In this work, we utilize the same process to develop a capacitive ethanol microsensor with a readout circuit on-a-chip. The sensitive material of the sensor is tin dioxide, because it has good sensitivity to ethanol gas [20,21]. The readout circuit converts the capacitance of the sensor into the frequency output. Dai *et al.* [22] presented a capacitive pressure sensor combined with a ring oscillator circuit and a micro-antenna as a wireless communication pressure sensor. The capacitance of the pressure sensor was converted by the ring oscillator into the frequency output, and then the frequency signal was transmitted using the micro antenna as a wireless signal. With the same method, the capacitive ethanol sensor has potential as a wireless communication ethanol sensor if combined with a micro-antenna.

## 2. Structure of the Ethanol Sensor

**Figure 1.**
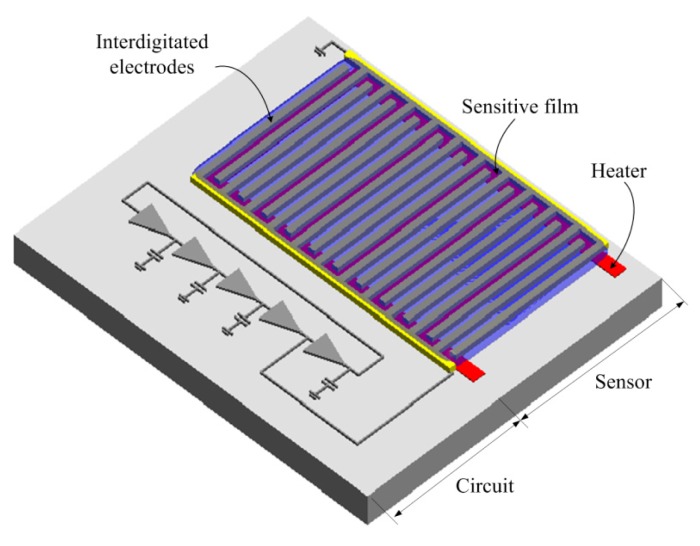
Structure of the integrated ethanol sensor.

The schematic structure of the ethanol sensor with a readout circuit is shown in Figure 1. The ethanol sensor contains a sensitive film, a heater and interdigitated electrodes. Tin dioxide is adopted as the sensitive film of the ethanol sensor. The sensitive film is deposited on the interdigitated electrodes, and the heater is set under the interdigitated electrodes. The material of the interdigitated electrodes is the aluminum metal of the CMOS process. The length, width and thickness of the electrodes are 230 μm, 10 μm and 6 μm, respectively, and the gap between the electrodes is 10 μm. The heater material is polysilicon. The heater is designed as a winding line, which it is utilized to supply a working temperature to the sensitive film. The dimensions of the heater are 2000 μm long, 30 μm wide and 0.2 μm thick. The ethanol sensor is a capacitive type. When the sensitive film adsorbs ethanol gas, the gas reacts with negative oxygen ions on the surface of tin dioxide [21], resulting in a capacitance change of the ethanol sensor.

**Figure 2.**
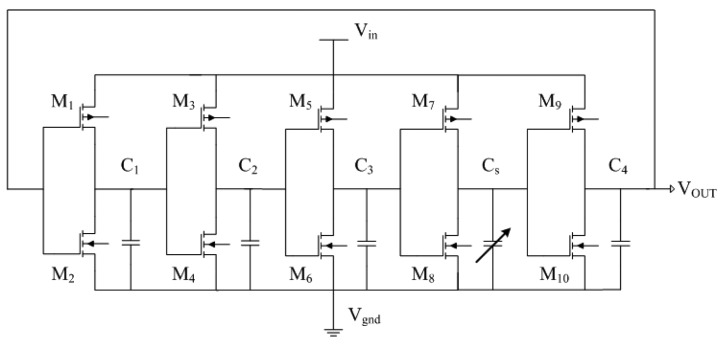
Readout circuit for the ethanol sensor.

A readout circuit is employed to convert the capacitance variation of the ethanol sensor into the output frequency. The readout circuit is a five-stage ring oscillator [23] as shown in Figure 2, where *M_1_, M_3_, M_5_, M_7_* and *M_9_* are p-channel metal oxide semiconductor (PMOS); *M_2_, M_4_, M_6_, M_8_* and *M_10_* are n-channel metal oxide semiconductor (NMOS); *C_s_* is the capacitance of the ethanol sensor; *C_1_, C_2_, C_3_* and *C_4_* are the load capacitance; *V_in_* is the input voltage; *V_ss_* is the ground; *V_out_* is the output voltage of the circuit.

**Figure 3.**
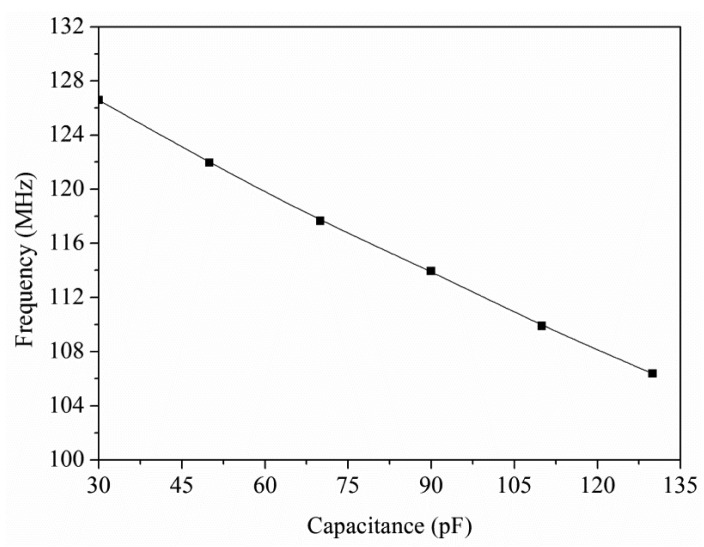
Simulated results of the output frequency for the circuit.

The output frequency of the ring oscillator circuit is simulated using the professional circuit simulation software, HSPICE (Synopsys Inc., Mountain, CA, USA). In the simulation, the load capacitance was 0.5 pF and the input voltage was 3 V. The ratio of width to length of all PMOS was 45:0.5, and the ratio of width to length of NMOS was 15:0.5. The capacitance of the ethanol sensor varied from 30 to 130 pF. Figure 3 presents the simulated results of the output frequency for the readout circuit. The simulated results depicted that the output frequency of the circuit decreased from 126.3 to 106.5 MHz as the capacitance changed from 30 to 130 pF.

## 3. Fabrication of the Ethanol Sensor

**Figure 4.**
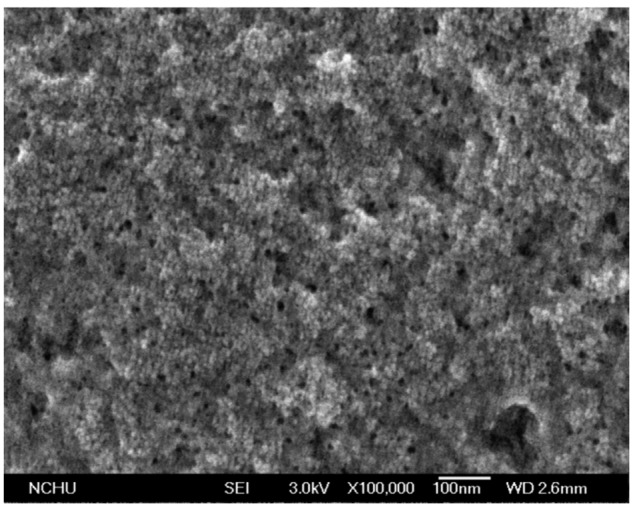
SEM image of the tin dioxide film.

**Figure 5.**
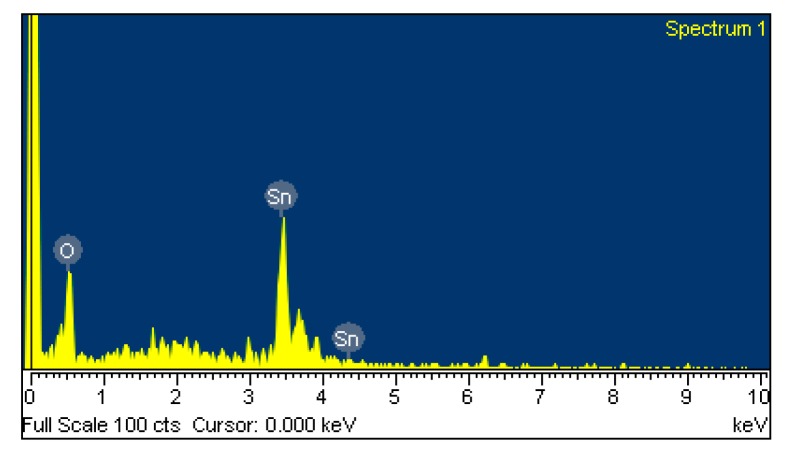
Elements of the tin dioxide film measured by EDS.

The sensitive film of the ethanol sensor was tin dioxide synthesized by a sol-gel method [24–26]. The tin dioxide was prepared as follows: stannic chloride (4 g) and glucose (4 g) were dissolved in deionized water (70 mL) with vigorous stirring until the solution was mixed uniformly. After the reaction, the resulting products were filtered, and washed with ethanol and deionized water. Finally, the tin dioxide was coated on the substrate, followed by calcination at 350 °C for 2 h. Scanning electron microscopy (SEM) [27] was used to measure the tin dioxide film. Figure 4 shows a SEM image of the tin dioxide film. The film has a porous structure that helps to increase its sensitivity. Energy dispersive spectrometry (EDS) [28] was utilized to analyze the composition of the tin dioxide film. Figure 5 presents elements of the tin dioxide film measured by EDS. The tested results showed that the film contained tin of 74.83 wt% and oxygen of 25.17 wt%.

**Figure 6.**
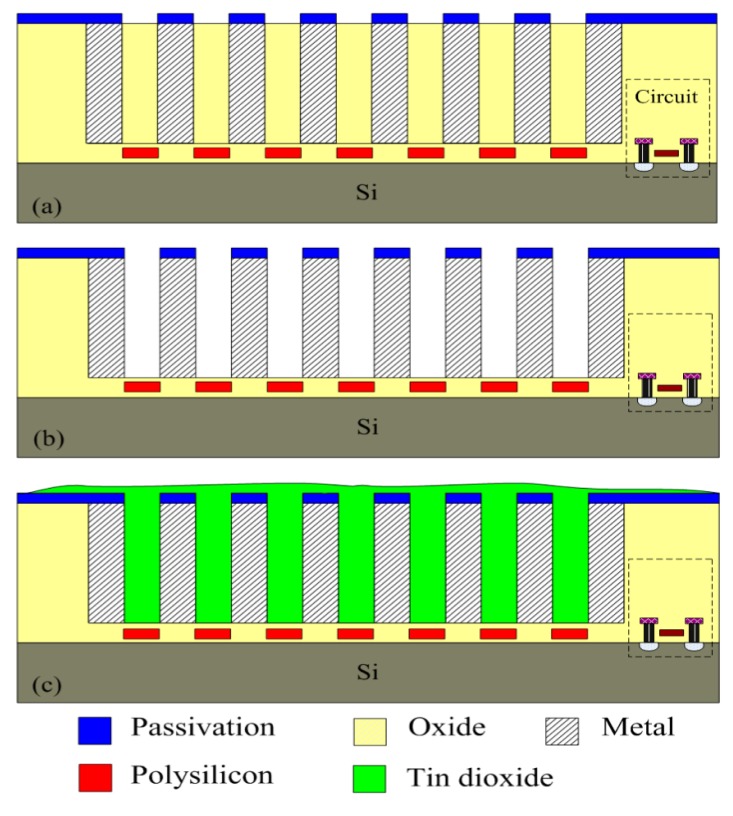
Fabrication process of the ethanol sensor: (**a**) after the CMOS process; (**b**) etching the sacrificial layer; (**c**) coating the tin dioxide film.

**Figure 7.**
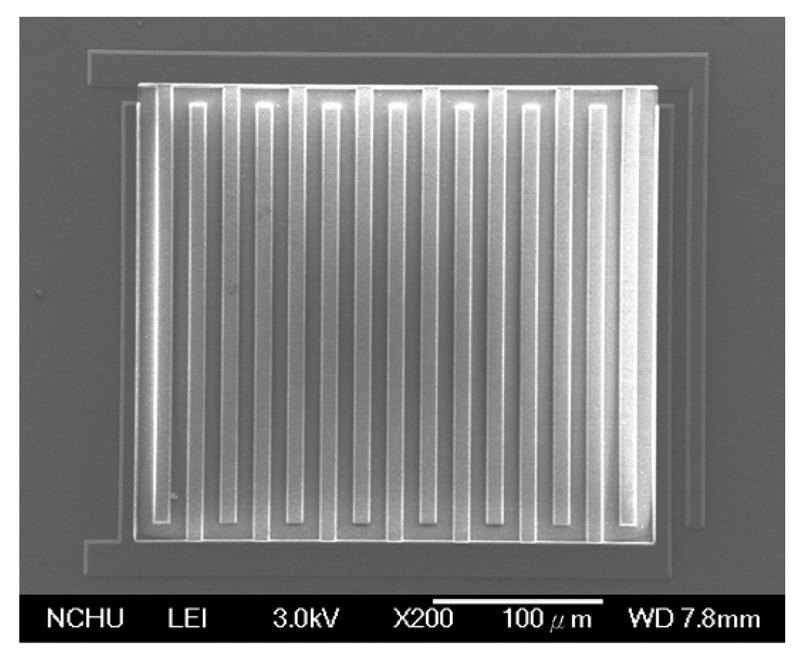
SEM image of the interdigitated electrodes after the wet etching.

**Figure 8.**
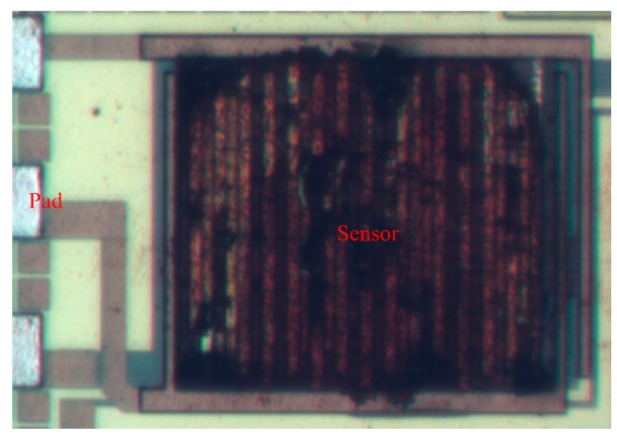
Optical image of the ethanol sensor after the post-process.

The integrated ethanol sensor chip was manufactured using the commercial 0.18 μm CMOS process of Taiwan Semiconductor Manufacturing Company (TSMC, Taipei, Taiwan). Figure 6 illustrates the fabrication flow of the ethanol sensor. Figure 6a shows the cross-sectional view of the ethanol sensor after the CMOS process. The interdigitated electrodes were formed by aluminum metal. The heater was made up of polysilicon. The oxide layer between the interdigitated electrodes was the sacrificial layer. The ethanol sensor required a post-process to deposit the tin dioxide film after the CMOS process. The post-process includes two main steps: (1) etching the sacrificial oxide layer; (2) depositing the tin dioxide onto the interdigitated electrodes. The sacrificial oxide layer is removed as shown in Figure 6b. A wet etching with buffer oxide etch (BOE) was employed to remove the sacrificial oxide layer [29,30], and to expose the interdigitated electrodes. The wet etching must be timed carefully to avoid undercutting the interdigitated electrodes. The etching rate of BOE was 960 Å/min [31]. The tin dioxide film is coated as shown in Figure 6c. A precision-control micro-dropper was used to drop the tin dioxide on the interdigitated electrodes, followed by the tin dioxide film was calcinated at 350 °C for 2 h. Figure 7 shows a SEM image of the interdigitated electrodes after the wet etching. Figure 8 shows an optical image of the ethanol sensor after the post-process.

## 4. Results and Discussion

A spectrum analyzer, a test chamber, a power supply, a LCR meter and an infrared thermometer were employed to measure the characteristics of the ethanol sensor. The LCR meter was used to detect the capacitance of the ethanol sensor, and the spectrum analyzer was utilized to record the output frequency of the ethanol sensor. The infrared thermometer measured the working temperature of the ethanol sensor. The test chamber included a control valve, a pump and a calibration ethanol sensor (BW GasAlertMicro5 PID, Honeywell Taiwan Ltd., Taipei, Taiwan). The calibration ethanol sensor could monitor *in-situ* the ethanol concentration in the test chamber. The control valve tuned the ethanol concentration in the test chamber, and the pump exhausted the ethanol gas in the test chamber.

**Figure 9.**
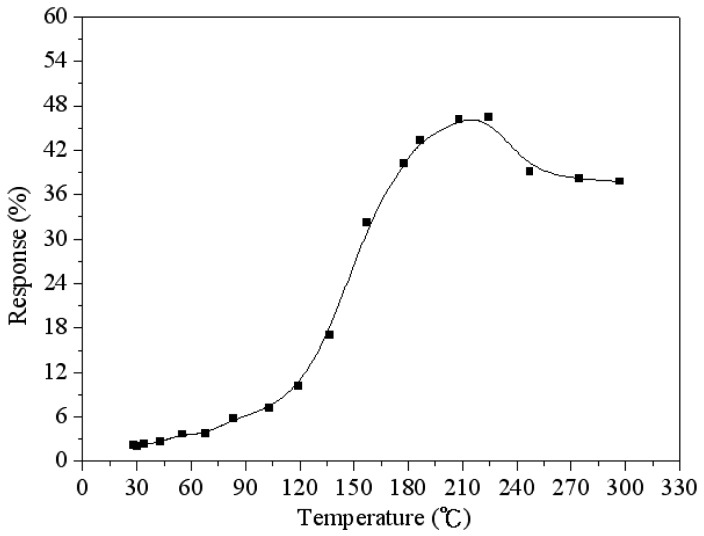
Response of the ethanol sensor at 3 ppm ethanol.

To characterize the optimum working temperature of the ethanol sensor, the sensor was tested under different temperatures. The ethanol sensor without the readout circuit was set in the test chamber. The control valve tuned ethanol gas to enter the test chamber, and the concentration of the test chamber was maintained at 3 ppm. The heater supplied different working temperatures to the ethanol sensor, and the LCR meter measured the capacitance of the sensor. Figure 9 shows the response of the ethanol sensor at 3 ppm ethanol. The response is defined as:

$$\left|\frac{C_{s}-C_{0}}{C_{0}}\right|\times 100$$

where *C_s_* is the capacitance variation of the ethanol sensor and *C_o_* represents the initial capacitance of the ethanol sensor at the beginning of all tests. The measured results showed that the optimum working temperature of the ethanol sensor was 220 °C.

**Figure 10.**
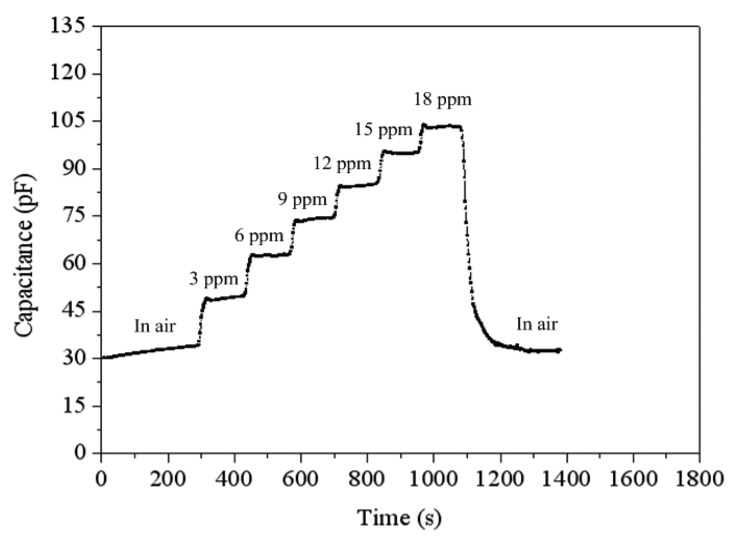
Test of the ethanol sensor.

**Figure 11.**
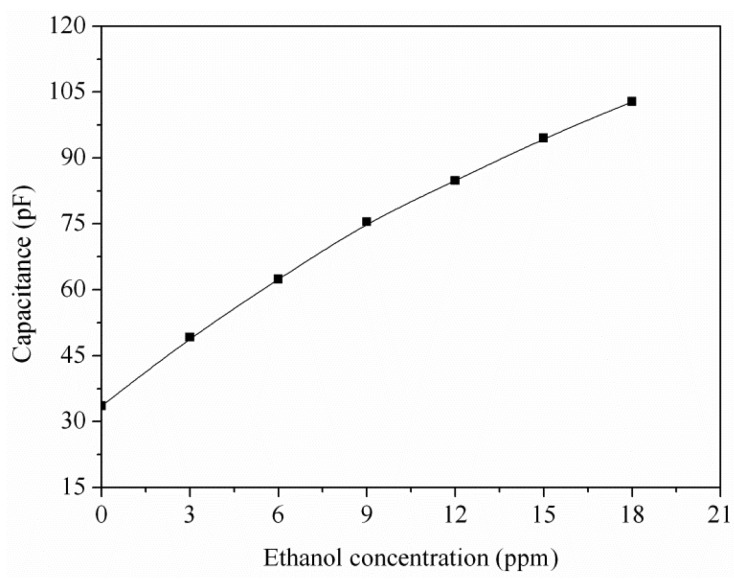
Capacitance variation of the ethanol sensor.

To understand the capacitance variation of the ethanol sensor, the sensor without the readout circuit was tested under different concentrations of ethanol at 220 °C (Figure 10). The LCR meter measured the capacitance variation of the sensor. The results revealed that the initial capacitance of the ethanol sensor was 34 pF in air, and the capacitance of the sensor increased to 102.8 pF at 18 ppm ethanol. The sensor recovered to the initial capacitance of 34 pF when it was in air. Figure 11 depicts the relation between the capacitance and ethanol concentration for the sensor. The results showed that the sensor had a capacitance of 49 pF at 3 ppm ethanol and a capacitance of 102.8 pF at 18 ppm ethanol. The capacitance of the sensor increased as the ethanol concentration increased.

**Figure 12.**
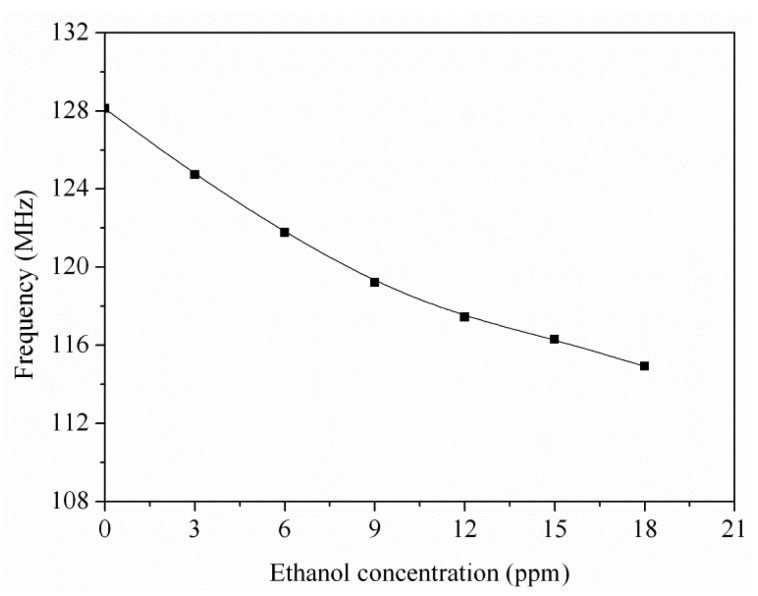
Measured results of the output frequency for the integrated ethanol sensor.

**Figure 13.**
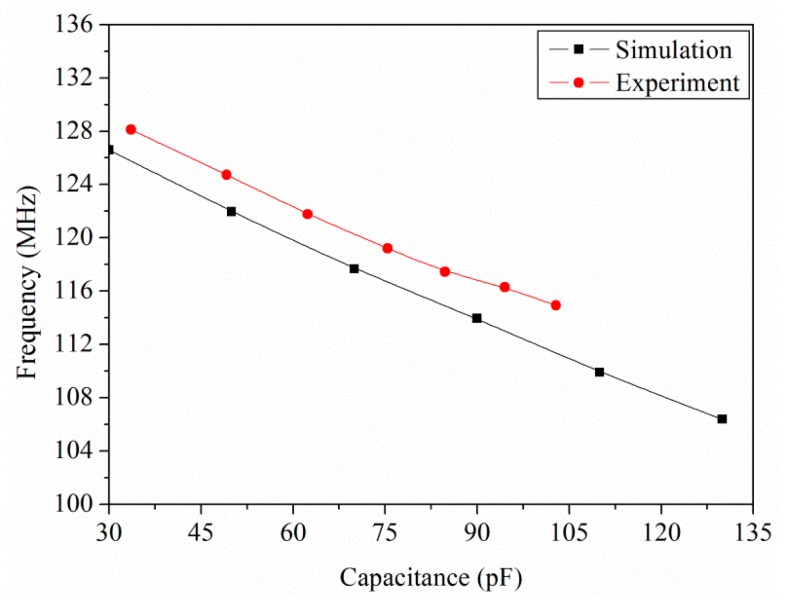
Relation between output frequency and capacitance for the sensor.

To characterize the output frequency of the integrated ethanol sensor, the sensor with the readout circuit was tested at different ethanol concentrations. The heater supplied a working temperature of 220 °C to the sensor. The power consumption of the sensor was 73 mW. The readout circuit converted the capacitance of the ethanol sensor into the output frequency. The power supply provided a bias voltage of 3 V to the circuit. The output frequency of the sensor was recorded by the spectrum analyzer. Figure 12 shows the output frequency of the integrated ethanol sensor. The measured results revealed that the output frequency of the ethanol sensor varied from 128.1 to 114.9 MHz as the concentration of ethanol gas increased from 0 to 18 ppm. The variation of the output frequency was 9 MHz in 0–10 ppm ethanol. Thereby, the ethanol sensor had a sensitivity of 0.9 MHz/ppm in ethanol range of 0–10 ppm. Figure 13 shows the relation between the output frequency and capacitance for the ethanol sensor, where the experimental results are given by combining the data in Figures 11 and 12, and the simulation results are the data in Figure 3.

**Figure 14.**
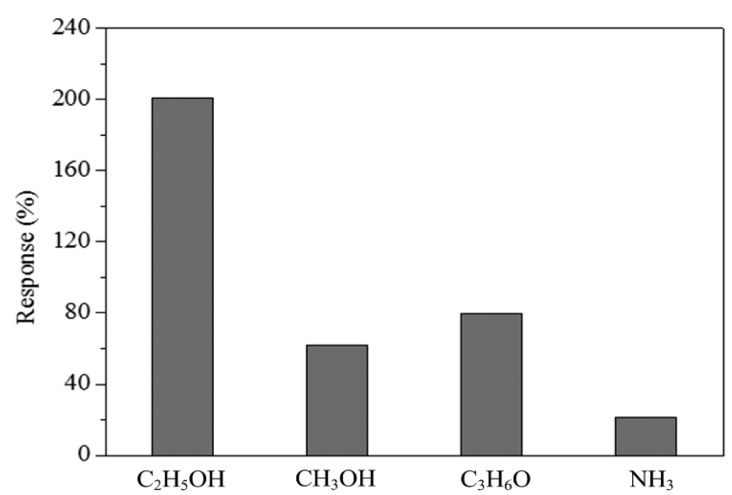
Response of the sensor under different gases.

As show in Figure 13, the experimental results approach to the simulation results and the percentage of error is under 2%. The sensor was tested with different gases in order to understand its selectivity. Figure 14 depicts the response of the sensor under ethanol, methanol, acetone and ammonia. In this investigation, the working temperature of the sensor was 220 °C, and the concentration of all gases was controlled at 15 ppm. The measured results revealed that the sensor for ethanol had a best response of 200%. Thereby, the sensor had an excellent selectivity for detecting ethanol.

## 5. Conclusions

An ethanol sensor with a readout circuit has been manufactured using the CMOS-MEMS technique. Tin dioxide was adopted as the sensitive material of the ethanol sensor, and it was prepared by a sol-gel method. In the post-processing, a BOE solution wet etching was employed to etch the sacrificial oxide layer between the interdigitated electrodes, followed by dropping the tin dioxide on the interdigitated electrodes. The tested results revealed that the optimum working temperature of the sensitive tin dioxide film for ethanol gas was 220 °C. The heater provided a working temperature of 220 °C to the sensitive film. The capacitance of the ethanol sensor changed as the sensitive film absorbed or desorbed ethanol gas. The readout circuit converted the capacitance of the sensor into the output frequency. The experimental results revealed that the capacitance of the ethanol sensor changed from 49 to 102 pF as the ethanol concentration increased from 3 to 18 ppm at 210 °C. The ethanol sensor with the readout circuit had a sensitivity of 0.9 MHz/ppm in ethanol range of 0–10 ppm.
